# Determinants of Self-Care in Diabetic Patients Based on Health Belief Model

**DOI:** 10.5539/gjhs.v7n5p33

**Published:** 2015-02-24

**Authors:** Abbasali Dehghani-Tafti, Seyed Saeed Mazloomy Mahmoodabad, Mohammad Ali Morowatisharifabad, Mohammad Afkhami Ardakani, Hassan Rezaeipandari, Mohammad Hassan Lotfi

**Affiliations:** 1 Department of Health Education & Promotion, Shahid Sadoughi University of Medical Sciences-Yazd, Iran; 2 Diabetes Research Center, Shahid Sadoughi University of Medical Sciences, Yazd, Iran; 3 Health Education, Elderly Health Research Center, Shahid Sadoughi University of Medical Sciences,Yazd, Iran; 4 Department of Epidemiology & Biostatistics, Shahid Sadoughi University of Medical Sciences-Yazd, Iran

**Keywords:** self-care, diabetes, health belief model, health education

## Abstract

**Introduction:**

The aim of this study was to determine self-care predictors in diabetic patients based on health belief model.

**Materials and Methods:**

The cross-sectional study was conducted on 110 diabetic patients referred to health service centers in Ardakan city, Yazd, Iran. The data was collected by a questionnaire including perceived benefits, barriers, severity, susceptibility, self-efficacy, social support, self-care behaviors and demographic variables.

**Results:**

Regularly medicine use (mean= 6.48 times per week) and shoes checking (mean= 1.17 times per week) were reported as the highest and the lowest self-care behaviors respectively. Health belief model constructs including perceived benefits, barriers, severity, susceptibility, self-efficacy and social support predicted 33.5% of the observed variance of self-care behaviors. Perceived susceptibility and self-efficacy had positive effect on self-care behavior; whereas perceived barrier’s has negative effect. Self-efficacy, perceived susceptibility and barriers were most powerful predictor respectively.

**Conclusion:**

The findings approved the efficiency of health belief model in prediction of self-care behaviors among diabetic patients. The findings realized the health belief model structure; therefore, it can be used as a framework for designing and implementing educational interventions in diabetes control plans.

## 1. Introduction

Diabetes is a common metabolic disorder and the fourth leading cause of death in western countries. Over 300 million people suffer from diabetes around the world. The World Health Organization estimated that diabetic patients in Iran will increase over 6 million people in 2030 (WHO, 2012). It is one of the major problems for human health which is related to urbanization and lifestyle changes such as inactivity and poor nutrition (Azizi, Janghorbani, & Hatami, 2010). Changes in lifestyle, industrialization, demographic and nutrition transition, obesity, inactivity and aging are considered among factors which are possible risk of diabetes in modern population (Landim, Zanetti, Santos, Andrade, & Teixeira, 2011). It is expected the numbers of patients increase over 500 million people during next 30 years (Javadi, Asgari, Yaghoobbi, & Tavazohi, 2010).

Diabetes imposes direct and indirect costs to the health service system and has negative influences on the quality of life. It is estimated that direct costs of diabetes include 2.5 to 15% of the annual budget allocated to health service system (Koo et al., 2011). Lack of self-care was identified as the most important reason of death in diabetics (Baquedano, Santos, Martins, & Zanetti, 2010). Lack of diabetes self-care behaviors can lead to increased complications of the disease (Jordan & Jordan, 2010). Self-care measures such as following a healthy diet, regular use of medications, regular exercise, and monitoring the blood glucose are proposed by International Diabetes Federation for optimal control of blood glucose (Peyrot & Rubin, 2007). Health care providers should promote diabetic patients self-care behaviors, using strategies such as health education programs (Karimy M, Abedi, Amin-Shokravi, & Tavafian, 2013). Despite simplicity of the most of self-care behaviors, many diabetics overlook these advises (Shakibazadeh et al., 2010). Regarding to close relation between healthy lifestyle and human behavior, behavioral based models can increase understanding about the factors influencing the health behaviors and determine their importance. Health Belief Model (HBM) that was selected as a theoretical framework for this study is one of the most effective models of health education which mainly focuses on disease prevention.

Previous studies showed successful application of HBM in explanation and prediction of preventive health behavior (Morowatisharifabad & Rouhani Tonekaboni, 2008). Based on HBM, individual must believe that he is susceptible to a disease (perceived susceptibility), understands the risk and it’s sever to his life (perceived severity), and follow the positive health behaviors such as self-care behavior (Shojaeizadeh, 2000). In this study HBM was used for determining the relationship between health beliefs and behavior toward diabetes in diabetic patients. The aim of this study was to determine factors influencing on self-care of diabetics patients based on HBM.

## 2. Materials and Methods

This is a cross- sectional study on 110 diabetic’s participants. The study population was diabetic patients with registered records in health care centers of health services network of Ardakan city, with diagnosed diabetes at least one year ago and being treated with medication (tablets or insulin). Sample size estimation was performed based on d=0.5and α=0.05 assumption, and the mean and standard deviation of self–care behaviors (m=36.4, SD=10.04) a previous study (Morowatisharifabad & Rouhani Tonekaboni, 2008). A researcher’s designed questionnaire was used for data collection. The first section consists of 21 questions on demographic and general characteristics of the patients such as age, education, sex, and marital status. The second part consists of 68 questions to measure HBM constructs. Content validity of the HBM questionnaire was confirmed by a panel of experts after conducting the necessary changes. To determine the internal consistency, a pilot study was conducted on 20 diabetic’s patients. Disabled or too old patients who were not able to understand the questions and answers, as well as those who were not aware of time and space were excluded. Data collection was performed in the health care centers and questionnaires randomly were completed. Patients who had been once studied, in the next few days were not included. They must be the resident of the city of our study (Ardakan) and consent to participate in the study.

The collected data were analyzed using SPSS statistical software. Pearson correlation coefficient test was used for examining the associations between the HBM constructs and self-care behaviors. Linear regression analysis was used for determining the prediction power of HBM constructs in explaining the variances of self-care behaviors. All null hypothesis were tested against 0.05 of significance level.

### 2.1 Measures

**Perceived benefits:** Seven questions of the Lewis and Bradley Scale (Bradley, 2013) were used to measure the perceived benefits. But to get a sense of accountability, the responses range was reduced to three parts from “disagree=1” to “agree= 3”. The grand score on this scale was computed by summing all questions score up and ranged between 7 and 21. The internal consistency of this scale in the pilot study and total population was α=0.693 and α=0.674 respectively.

**Perceived barriers:** 13 items scale of Becker, Stuifbergen, & Sands (Becker, Stuifbergen, & Sands, 1991) was used to measure perceived barriers. For every question we used a Likert type response of three grades from “none=1” to “many=3”. The grand score on this scale ranged between 13 and 39. Internal consistency of this scale in the pilot study and total population was α=0.652 and α=0.741 respectively.

**Perceived severity and susceptibility**: We used 13 Question of the Lewis and Bradley scale for perceived susceptibility and 15 question of that for perceived severity (Bradley, 2013). For each question we used a Likert of three grades from “none=1” to “many=3”. The grand score on perceived susceptibility was ranging between 13 and 39 and for perceived severity was in range of 15-45. Internal consistency of this two scale in a pilot study and the total population was α=0.741and α=0.787 for perceived susceptibility and α=0.826 and α=0.800 for perceived severity respectively.

**Perceived Self-efficacy:** To collect data on this scale we used self-care confidence scale of diabetes (CIDS) (Van der Ven et al., 2003) with a minor modification by Iranian experts and due to compliance with the study subjects. For every question we used a Likert of three grades of “none=1” to “many=3”. The grand score on this scale was in range of 20 to 60. Internal consistency of this scale in a pilot study and the total population was α=0.882 and α=0.827 respectively.

**Social support:** Schafer et al. scale of family support (Schafer, McCaul, & Glasgow, 1986) was used for social support measurement. Reliability and validity of the Persian version of the scale have been approved in the study of Morowatisharifabad et al. (Morowatisharifabad et al., 2007). The scale consists of 16 items, 9 items for measuring family supportive behaviors, and 7 items for family non-supportive behaviors. For responding the items a range of responses from “never=1” to “forever=5” was used. Scoring of non-supportive behavior was reverse. The range of scores on this scale was between 16 and 80.

**Self-care behaviors:** A 11 items Toobert & Glasgow scale of diabetes self-care was used (Toobert & Glasgow, 1994). Reliability and validity of the Persian version of the scale have been approved in a previous study (Morowatisharifabad et al., 2007). At this scale, attributes score was in range of 0 to 7. The total score was between 0 and 77.

## 3. Results

A total number of 110 diabetic patients were recruited from the medical centers of Ardakan city. The range of their ages was 26–75 with mean 55.2 (SD=10.66) years. About 85.5% of them were married. The education level of about 60% of participants was primary school, and only 8.2% of them had a university degree. 16.4% of the samples were office worker, 10 % blue collar worker, 22% unemployed and others were self-employed. Time of diagnosis of diabetes in the study population was between 2 to 35 years with a mean 11.04 ± 7.33 years.

Our results about the self-care behaviors showed, taking medicine regularly with average 6.48 times per week and inspect the inside of shoes with average 1.17 times per week was the highest and lowest reported self-care behaviors respectively. Consumption of fruits and vegetables and follow a healthy diet was reported for an average of 4.12 times in a week (Table 1).

**Table 1:** Frequency distribution of diabetes self-care behaviors among participants (n=110)

Number of behavior	0		1		2		3		4		5		6		7	
		
Kind of behavior in seven past days	N	%	N	%	N	%	N	%	N	%	N	%	N	%	N	%
		
In the past seven days, how many days you’ve pursued a healthy diet	0	0	2	1.8	9	8.2	26	23.6	15	13.6	19	17.3	18	16.4	21	19.1
In the past seven days, How many days a week did you follow your meal plan	2	1.8	11	10	12	10.9	14	12.7	22	20	18	16.4	18	16.4	11	11.8
In the past seven days how many day, did you used five size or more fruits and vegetables	1	0.9	0	0	14	12.7	43	39.1	25	22.7	20	18.2	7	6.4	0	0
In the past seven days how many time, used, fat foods such as red meat or high-fat dairy	1	0.9	20	18.2	26	23.6	28	25.5	25	22.7	7	6.4	3	2.7	0	0
In the past seven days at least 30 minutes of physical activity did you participate? (Ongoing activity, including walking)	6	5.5	30	27.3	32	29.1	21	19.1	8	7.3	10	9.1	1	0.9	2	1.8
In the past seven days How many times did you do a particular exercise sessions (such as swimming, walking, cycling), (Other than what you’ve done in your home or part of the work.)	42	38.2	31	28.2	16	14.5	7	6.4	8	7.3	5	4.5	1	0.9	0	0
In the past seven days How many times have checked your blood sugar	20	18.2	14	12.7	13	11.8	14	12.7	12	10.9	8	7.3	12	10.9	17	15.5
In the past seven days How many times cheeked your blood sugar frequently suggested by your doctor	19	17.3	13	11.8	9	8.2	7	6.4	7	6.4	14	12.7	20	18.2	21	19.1
In the past seven days how many times prescribed insulin (medication) as your doctor Saied	O	0	0	0	0	0	1	0.9	3	2.7	10	9.1	24	21.8	72	65.5
In the past seven days How many times have you checked your legs	17	15.5	22	20	8	7.3	23	20.9	15	13.6	12	10.9	10	9.1	3	2.7
In the past seven days How many times did you checked inside your shoes	39	35.5	38	34.5	18	16.4	8	7.3	6	5.5	0	0	0	0	1	0.9

Among the HBM constructs, the patients obtained the most scores in perceived severity. Also, they had high levels of scores in perceived benefits, while less than 50% of patients acquired self-care behavior scores. Table 2 shows the range, means and SDs of the constructs of HBM and self-care behaviors.

**Table 2:** Ranges, means and SDs of HBM constructs and self-care behaviors in diabetic patients (n=110)

Scores Structures of model	Possible score range	observed Range	Mean	SD	Percentage of score
Perceive benefits	7-21	12-21	18.38	2.18	87.5
Perceived barrier	13.39	18.35	26.1	3.68	66.92
Perceived severity	15-45	32-45	41.9	2.79	93.11
Perceived susceptibility	13-39	14-36	25.37	4.67	65.05
Self -efficacy	20-60	31-57	46.1	6.64	76.83
Social support	16.80	42-67	53.76	6.62	67.2
Self-care behavior	0-77	15-58	36.4	9.77	47.27

There was no significant difference between mean scores of self- care behaviors by sex and marriage, but there was a significant difference between some of HBM constructs and level of participant’s education. Based on the results of ANOVA with the Tukey post hoc test, average score of behavior and perceived social support in participants with primary education was significantly less than those with university degree.

There was a significant difference between the mean scores of self-care behaviors and perceived social support with job of participants. According to the Tukey post hoc test the mean scores of self-care behaviors and perceived social support of unemployed individuals was less than other occupational groups (Table 3).

**Table 3:** Means and SDs distribution of self-care behaviors scores and HBM constructs by demographic variables

constructs		Benefits		Barriers		Severity		susceptibility		Self-efficacy		Social Support		Behavior	

Variables		Mean	SD	Mean	SD	Mean	SD	Mean	SD	Mean	SD	Mean	SD	Mean	SD
Gender	Male	18.16	2.12	25.74	3.63	42.21	2.65	25.09	5.13	46.3	6.48	53.4	6.66	47.01	11.18
	Female	18.66	2.23	26.45	3.63	41.58	2.9	25.65	4.18	45.9	6.85	54.12	6.63	47.78	8.21
	P	0.296		0.315		0.233		0.530		0.754		0.567		0.684	
Education	Primary	18.7	2.33	26.24	3.67	41.78	2.86	25.2	4.45	44.83	6.72	52.55	6.35	45.33	9.54
	Guidance	18.45	1.46	26.2	4.31	41.1	3.27	26.9	4.22	49.2	5.2	57.45	6.63	52.45	6.42
	High school	19.37	1.74	26	3.7	43.3	1.7	24	5.5	46.68	6.32	53.12	6.54	45.75	8.82
	University	17.22	3.15	25	2.29	42	1.93	25.55	5.43	47.44	7.76	57.44	6.38	54	13.27
	P	0.108		0.822		0.117		0.291		0.063		0.026		0.004	
job	Office worker	19	2.44	25.38	3.66	42.38	1.91	24.55	5.12	48.27	6.27	55.22	6.32	49.61	12.04
	Worker	17.9	2.07	26.36	2.54	41.54	2.06	27	4.51	49.72	3.6	56.9	5.24	52.81	10.18
	Self-employed	18.29	1.9	25.03	3.61	42.45	3.1	25.64	5.14	45.58	6.65	53.38	6.46	48.12	9.76
	Unemployed	18	2.26	26.4	3.26	41.31	2.9	23.4	3.51	41.63	6.35	50.27	5.07	45.59	7.66
	Others	18.57	2.3	27.39	4.2	41.57	3.04	26.5	4.35	47.39	6.41	54.75	7.68	44.46	8.73
	p	0.581		0.138		0.517		0.109		0.002		0.034		0.098	

Pearson correlation test was used to investigate the relation between the HBM constructs and self-care behaviors. There was no correlation between self-care behaviors and perceived benefits, perceived barriers, perceived severity, but there was a positive significant correlation between self-care behaviors with perceived susceptibility, self-efficacy and social support (Table 4).

**Table 4:** Matrix of correlation coefficients of HBM constructs in a sample of diabetic patients (n=110)

Variable benefits barrier severity susceptibility self-efficacy social support behavior
Benefits 1
Barrier 0.95 1
Severity 0.341** 0.170 1
Susceptibility 0.231* 0.274** 0.160 1
Self-efficacy 0.209* 0.282** 0.115 0.680** 1
Social support 0.227* 0.360 ** 0.120 0.580** 0.288** 1
Self care behaviors -0.009 0.20 0.041 0.473** 0.491** 0.369** 1

* significant=0.05

** significant=0.01.

A stepwise linear regression analysis was used to examine the predictors of diabetes self-care behaviors by HBM constructs (Table 5). In general HBM constructs including perceived benefits, barriers, severity, self-efficacy, social support, totally predicted 33.5 percent of the observed variance in self-care behaviors. However, only the unique contribution of self-efficacy, susceptibility and barriers on observed variance of self-care behaviors were statistically significant. Self-efficacy was the strongest predictor of self-care behavior and by itself predicted 24.1% of observed variance in the model. The unique contribution of barriers, benefits, severity, and social support on self-care behaviors were not statistically significant.

**Table 5:** Results of stepwise regression analysis of predictors of diabetes self-care behaviors based on HBM (N=110)

Model	R Square	Std. Error of the Estimate	Change Statistics		

			R Square Change	F Change	Sig. F Change
A	.241	8.55216	.241	34.364	.000
B	.277	8.38709	.036	5.293	.023
C	.312	8.22256	.035	5.325	.023
D	.330	8.15023	.018	2.890	.092
E	.333	8.17083	.003	.471	.494
F	.335	8.19950	.002	.274	.602

A. Predictors: (Constant), self-efficacy; B. Predictors: (Constant), self-efficacy, susceptibility; C. Predictors: (Constant), self-efficacy, susceptibility, barriers; D. Predictors: (Constant), self-efficacy, susceptibility, barriers, benefits; E. Predictors: (Constant), self-efficacy, susceptibility, barriers, benefits, severity; F. Predictors: (Constant), self-efficacy, susceptibility, barriers, benefits, severity, social support.

## 4. Discussion

The results of this study showed that regular use of oral medication and insulin with 6.48 and check the shoes with 1.17 times in seven past days were the highest and lowest self-care behaviors. These results is in favor of the results of Nwasurba et al, that 85% of patients adhere to their medication regimen at all times of the week and 58% of them only once a day to control blood sugar (Nwasuruba, Khan, & Egede, 2007). The results of a study in Bandar-Abas-Iran revealed that patient acceptance and compliance in foot care was high, and 82% of patients, examined their feet daily and care them (Aghamollaei et al., 2003), that is not consistent with our results. However, in another study in Kermanshah, the mean score of foot care was lower than moderate before intervention, but after the educational intervention it significantly was increased (Sharifirad, Hazavehei, Mohebi, Rahimi, & Hasanzadeh, 2006). This finding is consistent with prior results of Sharifirad study and the results of after the intervention, emphasize the necessity and importance of foot care for diabetic patients and educational program for them. The reason for lack of foot care in diabetics of Ardekan is perhaps because of the use of open and flip-flap type shoe during the years, or culture of the using these shoes in daily activities.

The results of the present study showed that patients followed a healthy diet for an average of 4.61 times a week and 3.63 times in a week consumed the fruit and vegetable. Also, an average of 2.81 times a week have used fatty foods. That seems pretty high fat food regimen specifically for diabetic patients is considered but follow a healthy eating plan to meet the energy needs of their body is less attention, that must be considered in education of self-care in patients with diabetes.

The data of Parham et al. (Parham, Riahin, Jandaghi, & Darivandpour, 2013) revealed that 62.2% of patients used fruits and vegetables for 5 times in a day in the past week, 93.6% of them used carbohydrates in all days of the past week and only 8% of them avoided the entire meal of high fat and high-fat dairy foods. These findings are consistent with our results, therefore educating diabetes patients by nutritionist for dietary consultation is recommended.

The relative grand scores of participants in HBM constructs was 93.11%in perceived severity, 87.5% in perceived benefits, 76.83% in perceived self-efficacy, 65.05% in perceived susceptibility, and 66.92% in perceived barriers. The total score for behavior was lower than 50% (47.27%). The results of Shakibazadeh et al study on 128 type 2 diabetics patients, revealed that the practice of patients in self-care was poor (Shakibazadeh et al., 2009). Mahmoudi also found the same results (Mahmoudi, 2006). Some studies in developed countries showed that the self-care in those countries is better, but their practice is unsuitable (Vermeire et al., 2005). Some studies show a different impact of self-care behaviors on glyscemic control of their disease. Anbari et al. (Anbari KH, Kaviani, & Montazeri 2012) showed that the self -care of patients who lived with their wives and their children was better than those who are single or living with their children. These results is similar to the results of Lewko et al. (Lewko et al., 2007) in Poland and shows that the network quality of life of the patient and his social interactions with family members and emotional and psychological support to families on various issues had a positive effect on self-care behaviors. So it seems that more than half of the factors influencing the behavior should be acquired through a qualitative study with regard to other social phenomena, including factors related to the social network.

Our data showed that there was no significant difference between sex and self-care, that is similar to Liang et al. (Liang, Tsan, Ma, Chow, & Wu, n.d.), Pani et al. (Pani et al., 2008) and Charuruks et al. (Charuruks, Milintagas, Watanaboonyoungcharoen, & Ariyaboonsiri, 2005). There was no significant difference between mean grade scores of self-care and marriage of patients, that these results are same as data of Adwan et al. (Adwan & Najjar, 2013). The results of Trief et al revealed that there was no significant difference between marriage of patients and the rate of their blood Sugar. Therefore it seems that self-care behaviors are independent of these variables.

The results of present study showed significant difference between the mean grade scores of social support and self -care behavior with education of patients. Based on the results of the Tukey post hoc test, average score of behavior and perceived social support in patients with primary education was significantly less than university educated patients. It means that with increase in levels of education, self-care behaviors also will increases. These finding is consistent with results of Parham et al. (2013), Morowatisharifabad et al. (2007), Anbari et al. (Anbari KH et al., 2012; Lewko et al., 2007) and Karter et al. (2001). Higher level of education leads to the greater efficiency and the ability of diabetic patients to perform self-care behaviors. Some studies, such as Danai et al. (Danai, Tamadon, & Moonesan, 2004), reported no significant difference between the method of blood sugar control and education. But in the case of self-care behaviors with increasing levels of education, the promotion of health literacy with education is acceptable. In the case of high perceived social support among people with higher education there is necessary to find the reason in the community interaction of diabetic patients with society. The results of Carcone et al showed that perceived social support of families has a significant relationship with diabetes control among diabetic patients (Carcone, Ellis, Weisz, & Naar-King, 2011).

Mean score of perceived self-efficacy and social support in unemployed individuals was less than other occupational groups and as this group were 22% of the subjects it is an important point. It seems that unemployment not only may lose the support of those are around person, but also had some effect on self-steam and reduce feelings of efficacy over time in a person. The results of studies by Jafarian (Jafarian, Zabihi, Babaieasl, Eshkevari, & Bijani, 2010) and Parham (Parham et al., 2013) showed no significant difference between self-care behaviors and job of patients and their length of disease duration. The studies in USA showed that the impact of literacy in health is more than income and job. Illiteracy can lead to no feelings of responsibility for health care (Karimy M et al., 2013; Karimy, Montazeri, & Araban, 2012). As expected, there was a significant positive correlation between self-care behaviors and perceived susceptibility, perceived self-efficacy and perceived social support in this study, which is consistent with other studies (Morowatisharifabad et al., 2007; M. A. Morowatisharifabad & Rouhani Tonekaboni, 2008; Parham et al., 2013). In our study, HBM constructs overally predicted 33.5% of the variance in self-care behaviors. We found that, self –efficacy was the strongest predictor of self-care behavior among HBM constructs and by itself predicted 24.1% of observed variance in the model. However, perceived barriers, and perceived susceptibility were also significant predictors of self-care behavior in diabetic patients. It seems that focus on self–efficacy is the most influential element in the success of such health promotion programs. These findings could be valuable for planning of educational and other health promotion innervations in the case of diabetes management. Our findings suggests that the importance of benefits, severity, and social support on self-care behaviors is not comparable with self- efficacy, susceptibility and barriers. This rate in Morowatisharifabad et al. study was 45.3% (Morowatisharifabad et al., 2007) and in Gillibrand was 12% (Gillibrand & Stevenson, 2006). In the study by Aalto (Aalto & Uutela, 1997) 14% of the variance in adherence to dietary patterns, and 21 % of the variance in adherence to self-monitoring of blood glucose was predicted. The data of Brownlee (Brownlee-Duffeck et al., 1987) by using HBM, predicted 52 % of the variance in adherence to dietary.

in general, it can conclude that HBM constructs is a good fit to the data of this study and can be applied in the design of educational interventions for patients with diabetes. In several studies (Aljasem, Peyrot, Wissow, & Rubin, 2001; Glasgow, Toobert, & Gillette, 2001; Krichbaum, Aarestad, & Buethe, 2003; Morowatisharifabad & Rouhani Tonekaboni, 2008; Norris, Engelgau, & Narayan, 2001) self-efficacy was found as an important predictor of self-care behaviors in diabetes. A few studies showed that perceived barriers are the most influential predictor of self-care behaviors (Cameron, Worrall-Carter, Driscoll, & Stewart, 2009; Osborn & Egede, 2010). It should be noted that the lack of perceived social support is as one of the main obstacles to self- care, that here are shown as the perceived barriers, but at the correlation analysis between variables, social support has shown its role in the form of social support.

Limitations of the study: The findings of this study should be considered with its limitations. Use of self-report measures and methods of sampling which was a convenience sampling are among the possible limitation of the study. The sample restrictions create limitations on the generalizability of the findings. Given the importance of the factors on self-care that play an important role in preventing the complications of diabetes, more detailed studies and qualitative methods are necessary.

## 5. Conclusion

The findings of this study showed the relationship between the HBM constructs with self- care. Diabetic patients whatever are respect for self-care doing more relevant behavior. The higher susceptibility increases the behaviors of patients regardless of their barriers. Due to the increasing prevalence of diabetes and the importance of it for public health authorities to prevent complications of the disease, it is essential to promote self-care via enough training and sensitization of patients and their families about diabetes. Coordination and cooperation between the relevant health departments could be caused to minimize the barriers to self-care. It seems that the integration of diabetes intervention programs in chronic disease care model (CCM) can improve diabetes self-care.
